# Crystal structure of triclopyr

**DOI:** 10.1107/S160053681401681X

**Published:** 2014-08-01

**Authors:** Seonghwa Cho, Jineun Kim, Youngeun Jeon, Tae Ho Kim

**Affiliations:** a Department of Chemistry and Research Institute of Natural Sciences, Gyeongsang National University, Jinju 660-701, Republic of Korea

**Keywords:** crystal structure, herbicide, triclopyr, hydrogen-bonded dimers, π–π inter­actions, non-merohedral twinning

## Abstract

In the title compound {systematic name: 2-[(3,5,6-tri­chloro­pyridin-2-yl)­oxy]acetic acid}, the herbicide triclopyr, C_7_H_4_Cl_3_NO_3_, the asymmetric unit comprises two independent mol­ecules in which the dihedral angles between the mean plane of the carb­oxy­lic acid group and the pyridyl ring plane are 79.3 (6) and 83.8 (5)°. In the crystal, pairs of inter­molecular O—H⋯O hydrogen bonds form dimers through an *R*_2_^2^(8) ring motif and are extended into chains along [100] by weak π–π inter­actions [ring centroid separations = 3.799 (4) and 3.810 (4) Å]. In addition, short inter­molecular Cl⋯Cl contacts [3.458 (2) Å] connect the chains, yielding a two-dimensional architecture extending parallel to (020). The crystal studied was found to be non-merohedrally twinned with the minor component being 0.175 (4).

## Related literature   

For information on the toxicity and herbicidal properties of the title compound, see: McMullin *et al.* (2011 ▶); Carney *et al.* (2007 ▶). For a related crystal structure, see: Smith *et al.* (1976 ▶). Non-merohedral twinning in the crystal was identified usinTwinRotMat within *PLATON* (Spek, 2009 ▶).
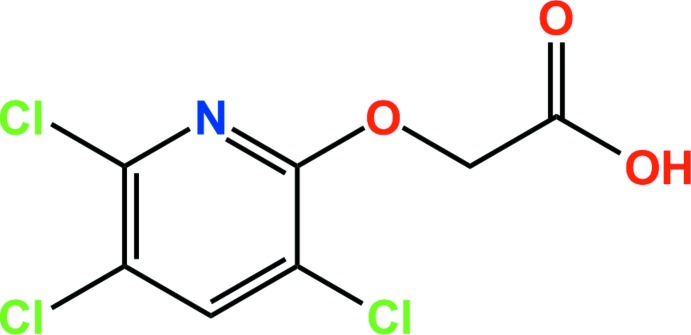


## Experimental   

### Crystal data  

C_7_H_4_Cl_3_NO_3_
*M* *_r_* = 256.46
Monoclinic, 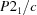

*a* = 7.5771 (9) Å
*b* = 25.409 (3) Å
*c* = 10.1668 (12) Å
β = 106.261 (8)°
*V* = 1879.1 (4) Å^3^
*Z* = 8
Mo *K*α radiation
μ = 0.95 mm^−1^
*T* = 173 K
0.50 × 0.09 × 0.06 mm

### Data collection  

Bruker APEXII CCD diffractometer
Absorption correction: multi-scan (*SADABS*; Bruker, 2009 ▶) *T*_min_ = 0.648, *T*_max_ = 0.945
3699 measured reflections
3699 independent reflections
3080 reflections with *I* > 2σ(*I*)
*R*_int_ = 0.000

### Refinement  

*R*[*F* ^2^ > 2σ(*F* ^2^)] = 0.044
*wR*(*F* ^2^) = 0.096
*S* = 1.12
3699 reflections
256 parameters
H-atom parameters constrained
Δρ_max_ = 0.32 e Å^−3^
Δρ_min_ = −0.28 e Å^−3^

### 

Data collection: *APEX2* (Bruker, 2009 ▶); cell refinement: *SAINT* (Bruker, 2009 ▶); data reduction: *SAINT*; program(s) used to solve structure: *SHELXTL* (Sheldrick, 2008 ▶); program(s) used to refine structure: *SHELXTL*; molecular graphics: *DIAMOND* (Brandenburg, 2010 ▶); software used to prepare material for publication: *SHELXTL*.

## Supplementary Material

Crystal structure: contains datablock(s) global, I. DOI: 10.1107/S160053681401681X/zs2309sup1.cif

Structure factors: contains datablock(s) I. DOI: 10.1107/S160053681401681X/zs2309Isup2.hkl

Supporting information file. DOI: 10.1107/S160053681401681X/zs2309Isup3.cml

**Fig. 1.**
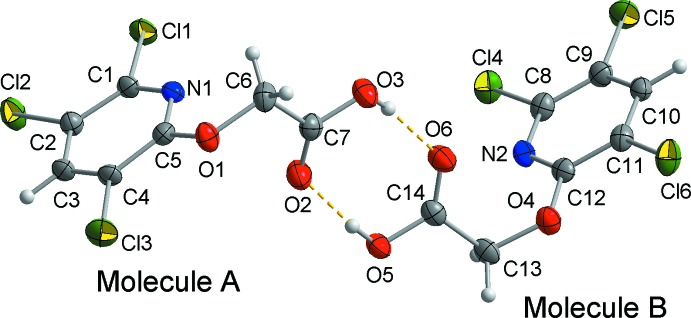
The asymmetric unit of the title compound with the atom numbering scheme. Displacement ellipsoids are drawn at the 50% probability level. H atoms are shown as small spheres of arbitrary radius.

**Fig. 2.**
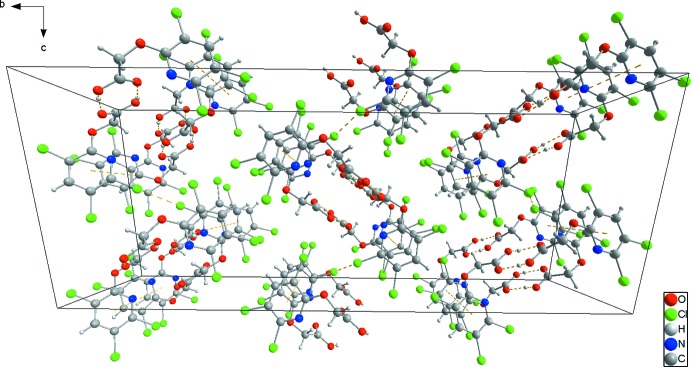
Crystal packing viewed along the a axis. The intermolecular O—H···O hydrogen bonds, weak π–π interactions, and short Cl···Cl contacts are shown as dashed lines.

. DOI: 10.1107/S160053681401681X/zs2309fig1.tif
The asymmetric unit of the title compound with the atom numbering scheme. Displacement ellipsoids are drawn at the 50% probability level. H atoms are shown as small spheres of arbitrary radius.

a . DOI: 10.1107/S160053681401681X/zs2309fig2.tif
Crystal packing viewed along the *a* axis. The inter­molecular O—H⋯O hydrogen bonds, weak π–π inter­actions, and short Cl⋯Cl contacts are shown as dashed lines.

CCDC reference: 1015180

Additional supporting information:  crystallographic information; 3D view; checkCIF report

## Figures and Tables

**Table 1:** Hydrogen-bond geometry (Å, °)

*D*—H⋯*A*	*D*—H	H⋯*A*	*D*⋯*A*	*D*—H⋯*A*
O3—H3*O*⋯O6	0.84	1.85	2.688 (3)	174
O5—H5*O*⋯O2	0.84	1.84	2.671 (3)	172

## supplementary crystallographic information

### S1. Comment

Triclopyr, C_7_H_4_Cl_3_NO_3_, is a herbicide used extensively in the control of woody plants and broadleaf weeds (McMullin *et al.*, 2011; Carney *et al.*, 2007), and its crystal structure is reported herein. In this compound (Scheme 1, Fig. 1). The asymmetric unit is composed of two independent molecules (Molecule A and Molecule B). The dihedral angles between the mean plane of the carboxyl groups and the pyridyl ring systems are 79.3 (6)° and 83.8 (5)° for Molecule A and Molecule B, respectively. All bond lengths and bond angles are normal and comparable to those observed in the crystal structure of a similar compound (Smith *et al.*, 1976).

In the crystal structure (Fig. 2), two carboxylic acid O—H···O hydrogen bonds are observed (Table 1), forming dimers through an *R*_2_^2^(8) ring motif. In addition, weak intermolecular π–π interactions between the pyridyl ring systems [*Cg*1···*Cg*2^i^, 3.799 (4) Å and *Cg*2···*Cg*1^ii^, 3.810 (4) Å], link the dimers into one-dimensional chains extending along (100) (*Cg*1 and *Cg*2 are the centeroids of the N1···C5 and N2···C12 rings, respectively). In addition, a short Cl···Cl contact [Cl1···Cl1^iii^, 3.458 (2) Å] is present [for symmetry codes: (i), -*x* + 1, -*y* + 1, -*z* + 1, (ii), -*x* + 2, -*y+* + 1, -*z* + 1, and (iii), -*x* + 1, -*y* + 1, -*z* + 2]. The crystal studied was found to be affected by non-merohedral twinning (Spek, 2009) and the data was treated accordingly, giving a final refined BASF parameter of 0.175 (4).

### S2. Experimental

The title compound was purchased from the Dr. Ehrenstorfer GmbH Company. Slow evaporation of a solution in CHCl_3_ gave single crystals suitable for X-ray analysis.

### S3. Refinement

All H-atoms were positioned geometrically and refined using a riding model with d(C—H) = 0.95 Å, *U*_iso_ = 1.2*U*_eq_(C) for aromatic C—H, d(C—H) = 0.99 Å, *U*_iso_ = 1.2*U*_eq_(C) for C*sp*^3^—H, and d(O—H) = 0.84 Å, *U*_iso_ = 1.5*U*_eq_(C) for O—H groups. Non-merohedral twinning in the crystal was identified [TwinRotMat within PLATON (Spek, 2009)]: [twin law -1 0 0, 0 -1 0, 0.751 0 1] giving a final refined BASF parameter of 0.175 (4).

### Crystal data

**Table:**

C_7_H_4_Cl_3_NO_3_	*F*(000) = 1024
*M**_r_* = 256.46	*D*_x_ = 1.813 Mg m^−^^3^
Monoclinic, *P*2_1_/*c*	Mo *K*α radiation, λ = 0.71073 Å
Hall symbol: -P 2ybc	Cell parameters from 3761 reflections
*a* = 7.5771 (9) Å	θ = 2.2–25.5°
*b* = 25.409 (3) Å	µ = 0.95 mm^−^^1^
*c* = 10.1668 (12) Å	*T* = 173 K
β = 106.261 (8)°	Needle, colourless
*V* = 1879.1 (4) Å^3^	0.50 × 0.09 × 0.06 mm
*Z* = 8	

### Data collection

**Table:**

Bruker APEXII CCD diffractometer	3699 independent reflections
Radiation source: fine-focus sealed tube	3080 reflections with *I* > 2σ(*I*)
Graphite monochromator	*R*_int_ = 0.000
φ and ω scans	θ_max_ = 26.0°, θ_min_ = 1.6°
Absorption correction: multi-scan (*SADABS*; Bruker, 2009)	*h* = −9→8
*T*_min_ = 0.648, *T*_max_ = 0.945	*k* = −31→31
3699 measured reflections	*l* = −5→12

### Refinement

**Table:**

Refinement on *F*^2^	Primary atom site location: structure-invariant direct methods
Least-squares matrix: full	Secondary atom site location: difference Fourier map
*R*[*F*^2^ > 2σ(*F*^2^)] = 0.044	Hydrogen site location: inferred from neighbouring sites
*wR*(*F*^2^) = 0.096	H-atom parameters constrained
*S* = 1.12	*w* = 1/[σ^2^(*F*_o_^2^) + (0.0219*P*)^2^ + 2.0682*P*] where *P* = (*F*_o_^2^ + 2*F*_c_^2^)/3
3699 reflections	(Δ/σ)_max_ = 0.001
256 parameters	Δρ_max_ = 0.32 e Å^−^^3^
0 restraints	Δρ_min_ = −0.28 e Å^−^^3^

### Special details

**Table:**

Geometry. All e.s.d.'s (except the e.s.d. in the dihedral angle between two l.s. planes) are estimated using the full covariance matrix. The cell e.s.d.'s are taken into account individually in the estimation of e.s.d.'s in distances, angles and torsion angles; correlations between e.s.d.'s in cell parameters are only used when they are defined by crystal symmetry. An approximate (isotropic) treatment of cell e.s.d.'s is used for estimating e.s.d.'s involving l.s. planes.
Refinement. Refinement of *F*^2^ against ALL reflections. The weighted *R*-factor *wR* and goodness of fit *S* are based on *F*^2^, conventional *R*-factors *R* are based on *F*, with *F* set to zero for negative *F*^2^. The threshold expression of *F*^2^ > σ(*F*^2^) is used only for calculating *R*-factors(gt) *etc*. and is not relevant to the choice of reflections for refinement. *R*-factors based on *F*^2^ are statistically about twice as large as those based on *F*, and *R*- factors based on ALL data will be even larger.

### Fractional atomic coordinates and isotropic or equivalent isotropic displacement parameters (Å^2^)

**Table:**

	*x*	*y*	*z*	*U*_iso_*/*U*_eq_	
Cl1	0.66278 (13)	0.53311 (3)	0.94222 (8)	0.0336 (2)	
Cl2	0.86627 (14)	0.62564 (3)	1.13208 (9)	0.0397 (2)	
Cl3	0.67175 (14)	0.75251 (3)	0.69082 (10)	0.0408 (2)	
Cl4	1.00995 (14)	0.31553 (3)	0.50221 (9)	0.0380 (2)	
Cl5	0.79593 (15)	0.23214 (3)	0.28102 (10)	0.0432 (3)	
Cl6	0.66657 (14)	0.38875 (3)	−0.09150 (8)	0.0403 (2)	
O1	0.4722 (3)	0.65825 (8)	0.5624 (2)	0.0317 (5)	
O2	0.6886 (3)	0.58941 (10)	0.4787 (3)	0.0386 (6)	
O3	0.4491 (3)	0.53970 (9)	0.3695 (3)	0.0382 (6)	
H3O	0.5290	0.5231	0.3434	0.057*	
O4	0.9075 (3)	0.44645 (8)	0.1341 (2)	0.0315 (5)	
O5	0.9552 (3)	0.54570 (9)	0.3905 (2)	0.0316 (5)	
H5O	0.8784	0.5599	0.4250	0.047*	
O6	0.7219 (3)	0.49064 (9)	0.2971 (3)	0.0361 (6)	
N1	0.5733 (4)	0.60258 (10)	0.7479 (3)	0.0248 (6)	
N2	0.9432 (4)	0.38215 (10)	0.3000 (3)	0.0258 (6)	
C1	0.6641 (4)	0.59584 (11)	0.8776 (3)	0.0242 (7)	
C2	0.7565 (5)	0.63593 (12)	0.9613 (3)	0.0267 (7)	
C3	0.7598 (5)	0.68502 (11)	0.9024 (3)	0.0278 (7)	
H3	0.8254	0.7133	0.9552	0.033*	
C4	0.6673 (5)	0.69213 (11)	0.7674 (3)	0.0254 (7)	
C5	0.5714 (4)	0.64974 (12)	0.6938 (3)	0.0241 (7)	
C6	0.3859 (5)	0.61313 (13)	0.4863 (3)	0.0331 (8)	
H6A	0.2905	0.6247	0.4032	0.040*	
H6B	0.3251	0.5920	0.5430	0.040*	
C7	0.5265 (5)	0.57983 (12)	0.4455 (3)	0.0283 (7)	
C8	0.9176 (5)	0.33276 (12)	0.3324 (3)	0.0266 (7)	
C9	0.8219 (5)	0.29661 (12)	0.2378 (3)	0.0276 (7)	
C10	0.7423 (5)	0.31367 (12)	0.1045 (3)	0.0279 (7)	
H10	0.6717	0.2901	0.0376	0.033*	
C11	0.7668 (5)	0.36496 (12)	0.0706 (3)	0.0266 (7)	
C12	0.8738 (4)	0.39780 (11)	0.1722 (3)	0.0255 (7)	
C13	1.0054 (5)	0.48192 (12)	0.2385 (3)	0.0301 (8)	
H13A	1.0624	0.5100	0.1967	0.036*	
H13B	1.1048	0.4627	0.3053	0.036*	
C14	0.8768 (5)	0.50619 (12)	0.3117 (3)	0.0269 (7)	

### Atomic displacement parameters (Å^2^)

**Table:**

	*U*^11^	*U*^22^	*U*^33^	*U*^12^	*U*^13^	*U*^23^
Cl1	0.0447 (5)	0.0215 (4)	0.0343 (4)	−0.0055 (4)	0.0106 (4)	0.0039 (3)
Cl2	0.0511 (6)	0.0334 (4)	0.0276 (4)	−0.0038 (4)	−0.0006 (4)	0.0000 (3)
Cl3	0.0532 (6)	0.0246 (4)	0.0453 (5)	−0.0031 (4)	0.0153 (5)	0.0085 (4)
Cl4	0.0483 (6)	0.0327 (4)	0.0293 (4)	0.0081 (4)	0.0048 (4)	0.0055 (3)
Cl5	0.0617 (7)	0.0248 (4)	0.0466 (5)	−0.0052 (4)	0.0210 (5)	0.0019 (4)
Cl6	0.0517 (6)	0.0405 (5)	0.0244 (4)	0.0102 (4)	0.0035 (4)	0.0000 (3)
O1	0.0372 (14)	0.0290 (12)	0.0261 (12)	0.0025 (11)	0.0044 (11)	−0.0002 (9)
O2	0.0295 (15)	0.0436 (14)	0.0423 (15)	−0.0027 (12)	0.0092 (12)	−0.0138 (11)
O3	0.0304 (14)	0.0387 (14)	0.0430 (15)	−0.0022 (12)	0.0064 (12)	−0.0143 (11)
O4	0.0415 (15)	0.0223 (11)	0.0290 (12)	0.0005 (10)	0.0072 (11)	0.0008 (9)
O5	0.0290 (13)	0.0293 (12)	0.0355 (13)	−0.0032 (11)	0.0074 (11)	−0.0063 (10)
O6	0.0315 (15)	0.0296 (12)	0.0471 (15)	−0.0033 (11)	0.0110 (12)	−0.0094 (11)
N1	0.0244 (15)	0.0246 (13)	0.0270 (14)	−0.0031 (12)	0.0098 (12)	−0.0019 (11)
N2	0.0274 (16)	0.0230 (13)	0.0253 (14)	0.0030 (12)	0.0044 (12)	−0.0023 (11)
C1	0.0277 (18)	0.0185 (14)	0.0297 (17)	−0.0006 (13)	0.0136 (15)	0.0012 (12)
C2	0.0278 (19)	0.0281 (16)	0.0238 (16)	−0.0001 (14)	0.0066 (14)	−0.0012 (13)
C3	0.033 (2)	0.0186 (15)	0.0332 (18)	−0.0043 (14)	0.0117 (16)	−0.0043 (13)
C4	0.0314 (19)	0.0181 (14)	0.0301 (17)	0.0001 (14)	0.0141 (15)	0.0033 (12)
C5	0.0248 (18)	0.0252 (16)	0.0244 (16)	0.0021 (13)	0.0105 (14)	−0.0001 (12)
C6	0.031 (2)	0.0383 (19)	0.0274 (18)	0.0025 (16)	0.0038 (16)	−0.0071 (14)
C7	0.034 (2)	0.0296 (17)	0.0194 (16)	0.0003 (15)	0.0038 (15)	0.0009 (13)
C8	0.0269 (19)	0.0256 (16)	0.0279 (17)	0.0085 (14)	0.0089 (15)	0.0038 (13)
C9	0.0301 (19)	0.0227 (15)	0.0331 (18)	0.0020 (14)	0.0138 (16)	0.0015 (13)
C10	0.0268 (19)	0.0272 (16)	0.0305 (18)	−0.0004 (14)	0.0096 (15)	−0.0083 (13)
C11	0.0265 (18)	0.0297 (16)	0.0235 (16)	0.0066 (15)	0.0069 (15)	−0.0018 (13)
C12	0.0252 (18)	0.0210 (15)	0.0322 (18)	0.0063 (14)	0.0113 (15)	−0.0021 (13)
C13	0.032 (2)	0.0223 (15)	0.0353 (19)	−0.0010 (14)	0.0080 (16)	−0.0012 (14)
C14	0.031 (2)	0.0197 (15)	0.0267 (17)	0.0019 (15)	0.0028 (15)	0.0028 (13)

### Geometric parameters (Å, º)

**Table:**

Cl1—C1	1.725 (3)	N2—C12	1.319 (4)
Cl2—C2	1.722 (3)	N2—C8	1.325 (4)
Cl3—C4	1.725 (3)	C1—C2	1.385 (4)
Cl4—C8	1.728 (3)	C2—C3	1.387 (4)
Cl5—C9	1.721 (3)	C3—C4	1.367 (4)
Cl6—C11	1.720 (3)	C3—H3	0.9500
O1—C5	1.354 (4)	C4—C5	1.394 (4)
O1—C6	1.434 (4)	C6—C7	1.507 (5)
O2—C7	1.204 (4)	C6—H6A	0.9900
O3—C7	1.314 (4)	C6—H6B	0.9900
O3—H3O	0.8400	C8—C9	1.379 (4)
O4—C12	1.341 (4)	C9—C10	1.389 (4)
O4—C13	1.431 (4)	C10—C11	1.374 (4)
O5—C14	1.318 (4)	C10—H10	0.9500
O5—H5O	0.8400	C11—C12	1.397 (4)
O6—C14	1.208 (4)	C13—C14	1.513 (5)
N1—C1	1.317 (4)	C13—H13A	0.9900
N1—C5	1.317 (4)	C13—H13B	0.9900
			
C5—O1—C6	116.6 (2)	O2—C7—O3	125.0 (3)
C7—O3—H3O	109.5	O2—C7—C6	123.6 (3)
C12—O4—C13	117.9 (2)	O3—C7—C6	111.3 (3)
C14—O5—H5O	109.5	N2—C8—C9	122.8 (3)
C1—N1—C5	118.6 (3)	N2—C8—Cl4	116.2 (2)
C12—N2—C8	119.0 (3)	C9—C8—Cl4	120.9 (2)
N1—C1—C2	123.5 (3)	C8—C9—C10	118.1 (3)
N1—C1—Cl1	116.4 (2)	C8—C9—Cl5	122.1 (3)
C2—C1—Cl1	120.1 (2)	C10—C9—Cl5	119.7 (2)
C1—C2—C3	117.6 (3)	C11—C10—C9	119.3 (3)
C1—C2—Cl2	121.7 (2)	C11—C10—H10	120.4
C3—C2—Cl2	120.7 (2)	C9—C10—H10	120.4
C4—C3—C2	119.1 (3)	C10—C11—C12	118.1 (3)
C4—C3—H3	120.5	C10—C11—Cl6	121.3 (3)
C2—C3—H3	120.5	C12—C11—Cl6	120.5 (2)
C3—C4—C5	118.7 (3)	N2—C12—O4	120.4 (3)
C3—C4—Cl3	120.1 (2)	N2—C12—C11	122.5 (3)
C5—C4—Cl3	121.2 (2)	O4—C12—C11	117.0 (3)
N1—C5—O1	119.7 (3)	O4—C13—C14	110.5 (3)
N1—C5—C4	122.4 (3)	O4—C13—H13A	109.6
O1—C5—C4	117.9 (3)	C14—C13—H13A	109.6
O1—C6—C7	110.3 (3)	O4—C13—H13B	109.6
O1—C6—H6A	109.6	C14—C13—H13B	109.6
C7—C6—H6A	109.6	H13A—C13—H13B	108.1
O1—C6—H6B	109.6	O6—C14—O5	125.5 (3)
C7—C6—H6B	109.6	O6—C14—C13	122.9 (3)
H6A—C6—H6B	108.1	O5—C14—C13	111.5 (3)
			
C5—N1—C1—C2	0.8 (5)	C12—N2—C8—C9	0.3 (5)
C5—N1—C1—Cl1	−179.5 (2)	C12—N2—C8—Cl4	−179.1 (2)
N1—C1—C2—C3	−3.1 (5)	N2—C8—C9—C10	−3.0 (5)
Cl1—C1—C2—C3	177.2 (2)	Cl4—C8—C9—C10	176.4 (2)
N1—C1—C2—Cl2	177.6 (3)	N2—C8—C9—Cl5	178.1 (3)
Cl1—C1—C2—Cl2	−2.1 (4)	Cl4—C8—C9—Cl5	−2.5 (4)
C1—C2—C3—C4	2.3 (5)	C8—C9—C10—C11	2.3 (5)
Cl2—C2—C3—C4	−178.4 (3)	Cl5—C9—C10—C11	−178.8 (3)
C2—C3—C4—C5	0.5 (5)	C9—C10—C11—C12	0.8 (5)
C2—C3—C4—Cl3	−179.1 (3)	C9—C10—C11—Cl6	−178.3 (2)
C1—N1—C5—O1	−177.0 (3)	C8—N2—C12—O4	−175.5 (3)
C1—N1—C5—C4	2.3 (5)	C8—N2—C12—C11	3.1 (5)
C6—O1—C5—N1	−4.9 (4)	C13—O4—C12—N2	−5.8 (4)
C6—O1—C5—C4	175.7 (3)	C13—O4—C12—C11	175.5 (3)
C3—C4—C5—N1	−3.0 (5)	C10—C11—C12—N2	−3.6 (5)
Cl3—C4—C5—N1	176.6 (2)	Cl6—C11—C12—N2	175.5 (2)
C3—C4—C5—O1	176.4 (3)	C10—C11—C12—O4	175.0 (3)
Cl3—C4—C5—O1	−4.1 (4)	Cl6—C11—C12—O4	−5.9 (4)
C5—O1—C6—C7	−75.3 (3)	C12—O4—C13—C14	−80.9 (3)
O1—C6—C7—O2	3.1 (5)	O4—C13—C14—O6	11.0 (4)
O1—C6—C7—O3	−176.7 (3)	O4—C13—C14—O5	−168.6 (2)

### Hydrogen-bond geometry (Å, º)

**Table:**

*D*—H···*A*	*D*—H	H···*A*	*D*···*A*	*D*—H···*A*
O3—H3*O*···O6	0.84	1.85	2.688 (3)	174
O5—H5*O*···O2	0.84	1.84	2.671 (3)	172
