# Supplementary figures and images for: Crystal structure of triclopyr
Source: Acta Crystallogr Sect E Struct Rep Online. 2014 Aug 1;70(Pt 9):o940. doi: 10.1107/S160053681401681X (PMC4186132; doi:10.1107/S160053681401681X)

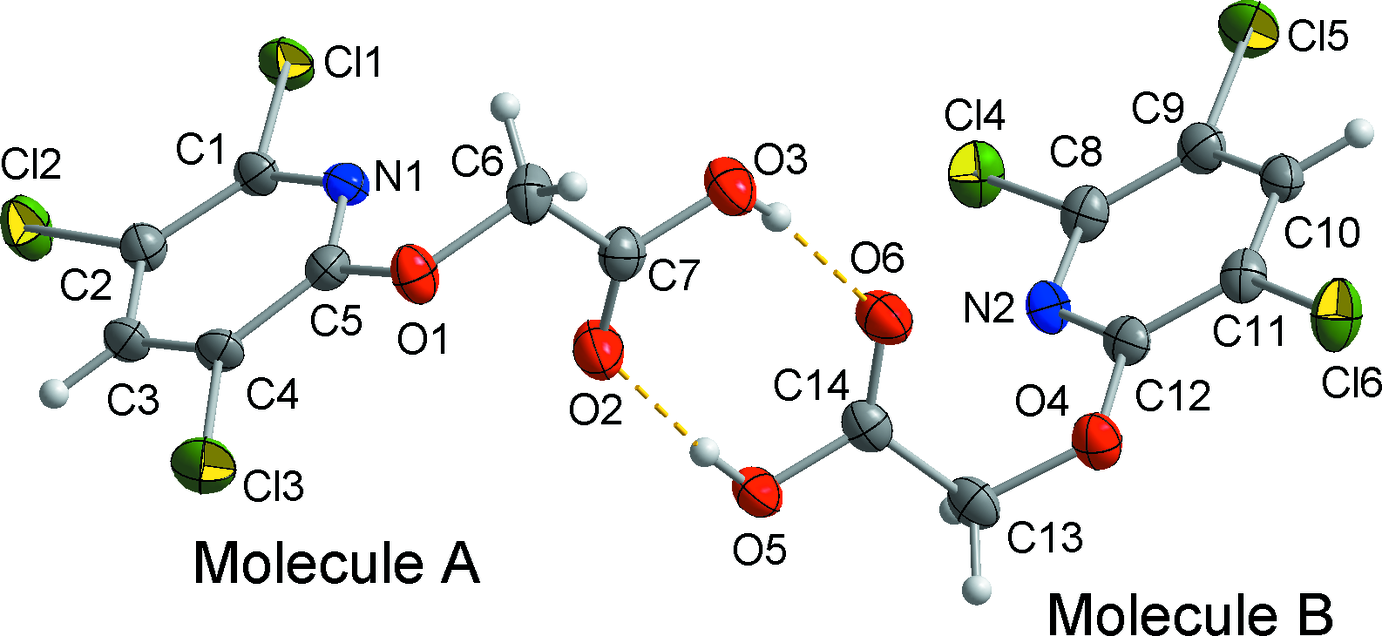

Supplement: Supplementary file 4 [file e-70-0o940-fig1.tif]

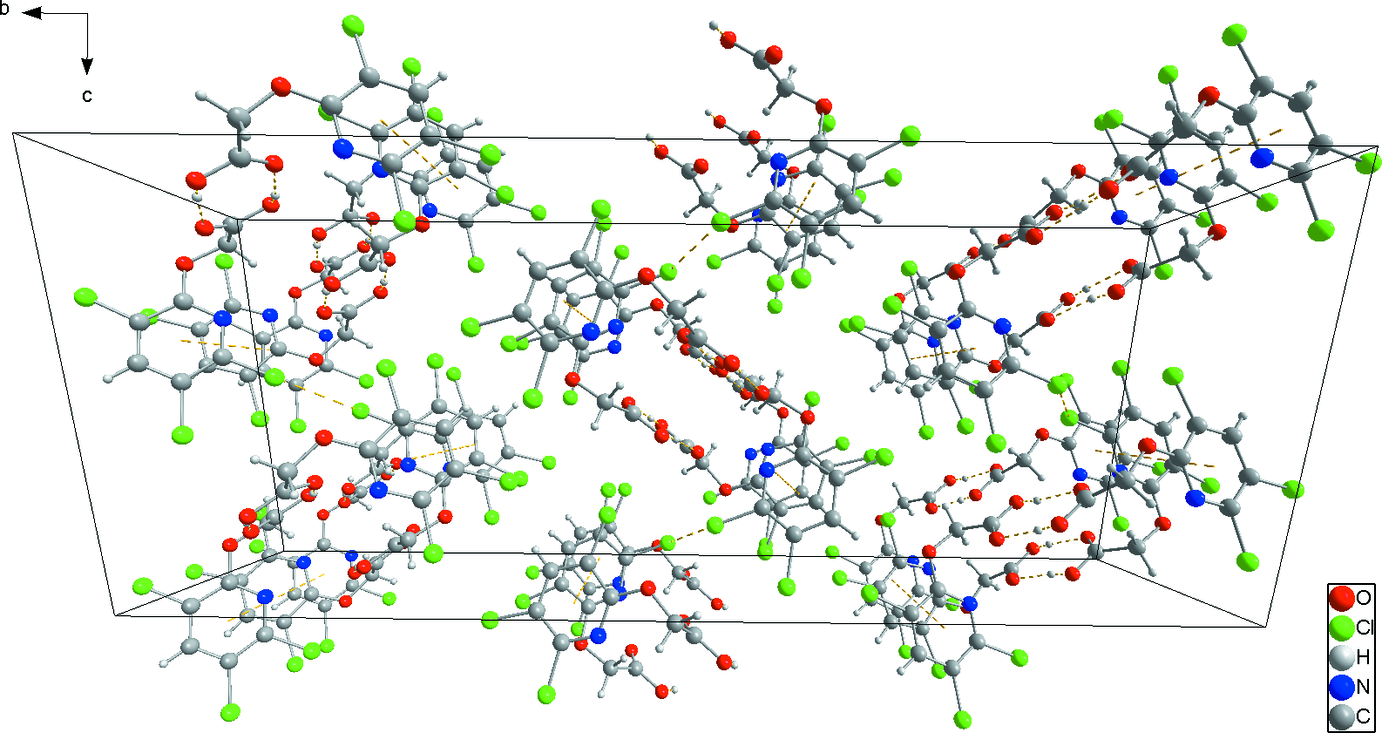

Supplement: Supplementary file 5 [file e-70-0o940-fig2.tif]
